# Novel Therapeutic Approaches for the Management of Elevated Lipoprotein(a): From Traditional Agents to Future Treatment Options

**DOI:** 10.3390/life14030374

**Published:** 2024-03-12

**Authors:** György Paragh, Péter Zilahi, László Róbert Kolozsvári, Hajnalka Lőrincz, Péter Fülöp, Mariann Harangi

**Affiliations:** 1 Division of Metabolism, Department of Internal Medicine, Faculty of Medicine, University of Debrecen, 4032 Debrecen, Hungary; 2 Department of Family and Occupational Medicine, Faculty of Medicine, University of Debrecen, 4032 Debrecen, Hungary

**Keywords:** lipoprotein(a), lipid-lowering therapy, lipoprotein apheresis, proprotein convertase subtilisin kexin type 9 inhibitor, antisense oligonucleotide, small interfering RNA

## Abstract

Cardiovascular disease is the leading cause of mortality worldwide. Despite the availability of effective low-density lipoprotein cholesterol (LDL-C) lowering agents, an increased cardiovascular risk is still observed in individuals with therapeutic LDL-C levels. One of these cardiovascular risk factors is elevated plasma lipoprotein(a) (Lp(a)) concentration, which maintains chronic inflammation through the increased presence of oxidized phospholipids on its surface. In addition, due to its 90 percent homology with the fibrinolytic proenzyme plasminogen, Lp(a) exhibits atherothrombotic effects. These may also contribute to the increased cardiovascular risk in individuals with high Lp(a) levels that previous epidemiological studies have shown to exist independently of LDL-C and other lipid parameters. In this review, the authors overview the novel therapeutic options to achieve effective Lp(a) lowering treatment, which may help to define tailored personalized medicine and reduce the residual cardiovascular risk in high-risk patients. Agents that increase LDL receptor expression, including statins, proprotein convertase subtilisin kexin type 9 inhibitors, and LDL production inhibitors, are also discussed. Other treatment options, e.g., cholesterolester transfer protein inhibitors, nicotinic acid derivatives, thyroid hormone mimetics, lipoprotein apheresis, as well as apolipoprotein(a) reducing antisense oligonucleotides and small interfering RNAs, are also evaluated.

## 1. Introduction

Lipoprotein(a) (Lp(a)) was discovered in 1963, and later studies confirmed that it increases cardiovascular risk [1]. Observational studies on large populations, as well as subgroup analyses of lipid-lowering treatments, found an exponential relationship between Lp(a) levels and the risk of myocardial infarction, stroke, and peripheral arterial disease [1,2,3,4,5]. Attention was also drawn to the fact that increased Lp(a) value increased the incidence of aortic valve stenosis, which was explained by the deposition of oxidized phospholipids in Lp(a), maintaining chronic inflammation in the valve. Lipoprotein-associated phospholipase A2 converts phospholipids into lysophosphatidylcholine, which increases the mineralization of the valves. Carried by the Lp(a) molecule, autotaxin helps to convert lysophosphatidylcholine into lysophosphatidic acid, which favors the production of osteoblast-directed transcription factor and bone morphogenetic protein-2 in the valvular interstitial cells [6]. As a result of these changes, osteoblast-like cells promote subsequent valvular calcification, as well [7]. The increased cardiovascular risk may partly be because apolipoprotein(a) (apo(a)) binds to low-density lipoprotein (LDL) and slows down its metabolism. As a result, increased cholesterol levels and high phospholipid content of the apo(a) protein can also be detected for a longer period of time, contributing to chronic inflammation.

**Figure 1:**
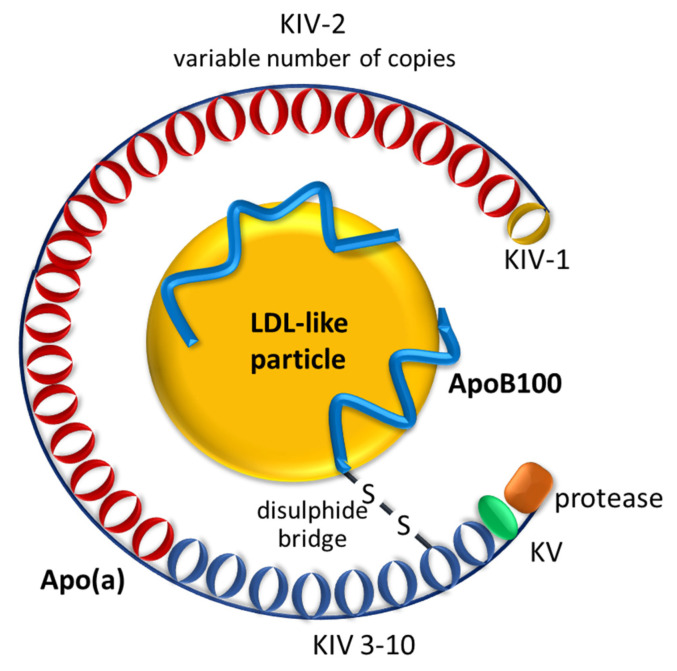
Structure of lipoprotein(a) (Lp(a)). Lp(a) consists of an LDL-like, lipid-rich particle with apolipoprotein(a) (apo(a)) on its surface. Apo(a) binds to apolipoprotein B100 (ApoB100) via a covalent disulfide bridge. Apo(a) contains repeated kringle (K) structures (KIV and KV) comparable with those in plasminogen. Numerous genetically determined apo(a) size isoforms have been described, which means different varying numbers of identical copies of kringle-IV type 2, resulting in the 300 kDa lowest molecular weights of the apo(a) phenotypes to 800 kDa [8].

The Lp(a) structure is similar to that of LDL regarding the size and lipid composition of the particles and the presence of apolipoprotein B100 (apoB100). The major structural difference between them is that, in addition to apoB100, Lp(a) has a second protein, apo(a), bound to apoB100 via noncovalent interactions and one single disulfide bridge (Figure 1) [8]. Examining the composition of the apo(a) protein, it was found that it is 90% homologous to the structure of plasminogen and inhibits the conversion of plasminogen to plasmin, as well as increases the production of plasminogen activator inhibitor-1 [9]. In addition, there are two other domains constituted by highly glycosylated, tridimensional heavy-chain structures known as kringles (K). Of the kringle domains of apo(a), one is similar to the kringle V (KV) of plasminogen. The other, kringle-IV (KIV), which is present only once in the plasminogen structure, has ten different types in apo(a) (KIV-1 to 10). Only KIV-2 occurs repeatedly in the apo(a) sequence (10 to 40 times) [8].

In the circulation, the Lp(a) serum level shows a close relationship with the weight of the molecule. In the case of the accumulation of low molecular weight apo(a), the serum level increases, while in the case of high molecular weight, a lower serum level should be expected. Therefore, precise analytical methods with high accuracy, reproductivity, selectivity, and sensibility are needed to determine Lp(a) quantification in serum. Manufacturers are offering several commercial laboratory test systems for Lp(a) measurement, including immunoturbidimetric or nephelometric systems applying polyclonal antibodies against apo(a). In addition, some manufacturers expressed a mass assay in mg/dL and a molar assay in nmol/L for Lp(a) [10,11]. One of the problems in the quantification of Lp(a) arises from the size polymorphism of apo(a) [12]. One may hypothesize that depending on the assay type and antibody specificity, which are largely unknown in the commercial assays, the size polymorphism of apo(a) may impact the results. For instance, the molar level of Lp(a) may be underestimated in individuals with small isoforms and high levels, while it may be overestimated in individuals with large isoforms and low levels when one uses a single reference standard [12]. Because of these methodological difficulties, there is no gold-standard method to determine Lp(a) concentration in the serum, and further consistent standardization procedures are needed. Also, clinicians should take these considerations into account for the patient therapy.

Thus, early and effective Lp(a)-lowering treatment is of major importance in reducing cardiovascular risk in individuals with high Lp(a) levels. Also, previous epidemiological studies have shown that high Lp(a) levels can be observed in about 20% of the European population [13]. It was also suggested that high levels of Lp(a) were more common in individuals with familial hypercholesterolemia, further increasing the cardiovascular risk in these patients [14]. On the other hand, the extremely large burden of vascular disease in FH patients is mainly explained by the high prevalence of clustered traditional risk factors, including the high prevalence of smoking, obesity, and hypertension [15].

**Figure 2:**
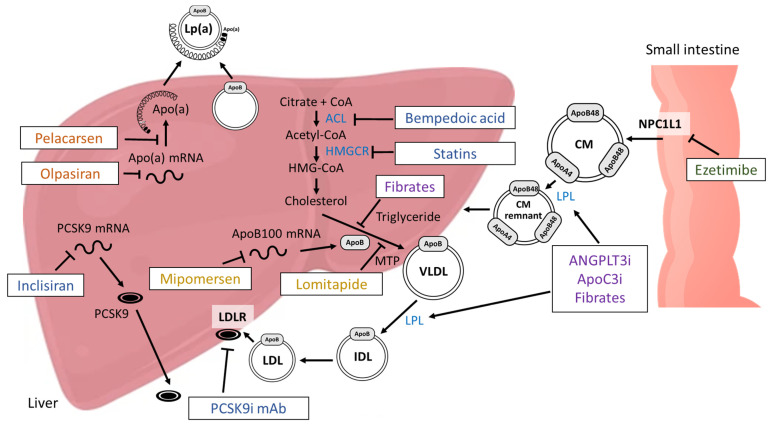
Mechanisms of action of the currently available lipid-lowering therapies. Bempedoic acid and statins prevent cholesterol synthesis by inhibiting adenosine triphosphate citrate lyase (ACL) and 3-hydroxy-3-methylglutaryl coenzyme reductase (HMGCR), respectively. Ezetimibe competitively inhibits the transport of sterols into enterocytes via Niemann-Pick C1-like 1 protein (NPC1L1). Fibrates prevent the synthesis of triglycerides and very low-density lipoprotein (VLDL) production. Angiopoetin-like 3 protein inhibitors (ANGPTL3i), fibrates, and ApoC3 inhibitors (apoC3i) improve lipoprotein lipase (LPL) activity. Monoclonal antibodies (mAb) against proprotein convertase subtilisin kexin type 9 (PCSK9) inhibit PCSK9 binding to low-density lipoprotein receptor (LDLR). Lomitapide inhibits microsomal triglyceride transfer protein (MTP), which prevents the formation of apolipoprotein B (apoB) and, thus, the formation of VLDL and chylomicrons (CM). Mipomersen targets the RNA encoding apolipoprotein B100 (apoB100) and reduces the production of the apoB100 protein. Inclisiran prevents the translation of PCSK9 mRNA. Pelacarsen inhibits the synthesis of apolipoprotein(a) (apo(a)). Olpasiran is a small interfering RNA molecule that markedly reduces lipoprotein(a) (Lp(a)) production in the hepatocytes by degrading apo(a) mRNA. CoA, coenzyme A; IDL, intermediate-density lipoprotein.

In the last few decades, numerous novel pharmaceutical agents have been developed to control and modify the composition of blood lipids to ultimately prevent fatal cardiovascular events in patients with dyslipidemia (Figure 2). In this work, we summarize the possibilities that can contribute to diminishing cardiovascular risk by reducing the Lp(a) level. It must be noted that some agents have more than one mechanism of action.

## 2. Agents That Increase LDL Receptor Expression

### 2.1. Statins

**Figure 3:**
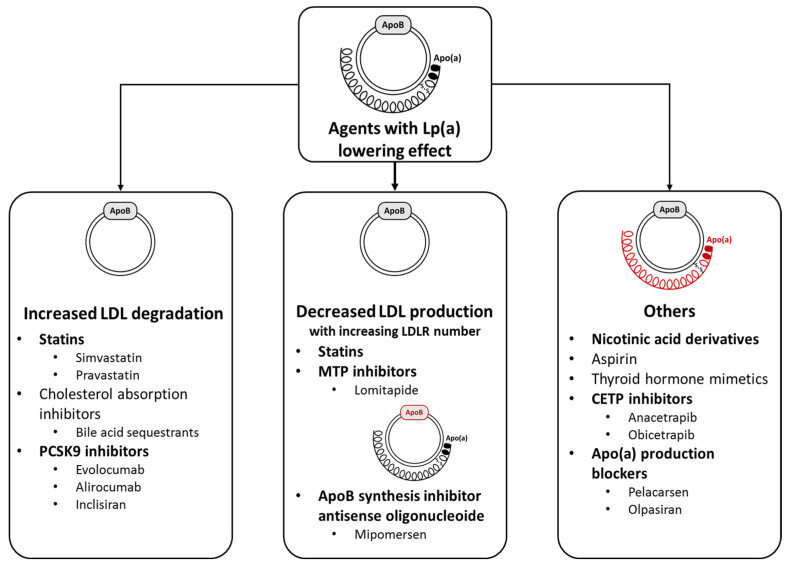
Agents with Lp(a) lowering effect according to their main mechanism of action. Abbreviations: Apo(a), apolipoprotein (a); ApoB, apolipoprotein B; CETP, cholesteryl ester transfer protein; LDL, low-density lipoprotein; LDLR, low-density lipoprotein receptor; Lp(a), lipoprotein(a); MTP, microsomal transfer protein; PCSK9, proprotein convertase subtilisin/kexin type 9.

Since apo(a) is covalently bound to the LDL particle, and this determines the Lp(a) level, it is tempting to speculate that drugs inducing increased LDL clearance affect Lp(a) levels. The most widely used such drugs are statins, which augment cholesterol uptake from the extracellular space by increasing the number of LDL receptors on the surface of cells. In the CARDS study, a 13% decrease in Lp(a) was observed with atorvastatin treatment [16], while the Scandinavian Simvastatin Survival Study (4S) found a 15% increase in Lp(a) with simvastatin treatment [17]. A meta-analysis of six randomized studies, including 5256 patients, indicated that statins moderately increased circulating Lp(a) concentrations [18]. This raised the possibility that the metabolism of Lp(a) takes place not only through the LDL receptor (LDLR) [19] but also through the LDL receptor-related protein 1, which can be modified by apoE isoforms or by CD36, via SR-B1 and plasminogen receptor [20]. Another question is how statins affect the formation of apo(a) protein and the expression of its mRNA. To clarify this, HepG2 cell cultures were incubated with various concentrations of statins, and mRNA levels of LDLR, PCSK9, and apo(a) were investigated [18]. Although statins uniformly increased LDLR expression on the surface of cells, they also augmented the formation of the apo(a) protein, which might contribute to the less efficacy of statins on Lp(a) levels. Drugs with Lp(a) lowering effect, according to their main mechanism of action are demonstrated in Figure 3.

### 2.2. Ezetimibe

If the LDL target value is not reached with the maximally tolerated dose of statins, ezetimibe represents a rational option as an add-on treatment to improve LDL reduction. Ezetimibe selectively prevents cholesterol absorption from the small intestine by inhibiting the Niemann-Pick C1-like protein and may lead to a 20% reduction of LDL-C levels. A meta-analysis of seven randomized, controlled trials found that Lp(a) levels decreased by 7.06% in patients with primary hypercholesterolemia upon the administration of ezetimibe [21]. On the other hand, a different meta-analysis concluded that ezetimibe did not significantly affect Lp(a) levels [22]. It is also important to mention that, even with the combined use of statin + ezetimibe, the target LDL-C levels are not attained in a remarkable number of patients with familial hypercholesterolemia (FH) [23].

### 2.3. PCSK9 Inhibitors

In light of the above-mentioned, one of the most important drug developments of the last decade was the procreation of PCSK9 inhibitors, which interfere with the destruction of the LDLR in the hepatocytes. The role of the PCSK9 protein is to bind to the LDLR and promote its breakdown within the cell. Physiologically, LDLR recirculates 100–150 times between the cell plasma and the cell surface [24,25]; the recirculation process is shortened by the PCSK9 protein, resulting in a decreased number of LDLRs on the surface of the hepatocytes [26]. Gain-of-function mutations showed increased PCSK9 activity, resulting in significantly higher cholesterol levels in the bloodstream and enhanced atherosclerosis [24]. On the contrary, cholesterol levels and atherosclerosis risk decreased in the case of loss-of-function mutations [27]. Given the structural homology between LDL and Lp(a), the LDLR has received the most attention as a candidate receptor for Lp(a), and the development of PCSK9 inhibitors began. After the statins and ezetimibe, PCSK9 inhibitors represent the next step in lipid-lowering therapy.

This class of drugs includes two large groups of medication, including the monoclonal antibodies evolocumab and alirocumab and the small interfering RNA (siRNA) inclisiran. An animal study on a murine model demonstrated that LDLR is not a route of Lp(a) plasma clearance since modulation of LDLR expression with alirocumab did not alter the cellular or the hepatic uptake of Lp(a) [28]. In the FOURIER study, at Lp(a) levels greater than the average 37 nmol/L, the incidence of cardiovascular death, acute myocardial infarction, urgent revascularization, and coronary disease increased. Administration of evolocumab resulted in a 23% decrease of Lp(a) in those with higher Lp(a) values, while there was a 7% decrease in the case of average Lp(a) levels [29]. In the ODYSSEY Outcome study, the effect of Lp(a) on clinical endpoints was investigated. Lp(a), independently from non-high-density lipoprotein (non-HDL) and LDL-cholesterol (LDL-C) levels, influenced cardiovascular complications [30]. The mean baseline Lp(a) value was 21.2 mg/dL. In the group with an average Lp(a) value, a 23.6% decrease in Lp(a) was observed, which contributed to a 14% reduction in major cardiovascular events. Shapiro et al. found that a 50–60% reduction in LDL with PCSK9 inhibitors was associated with a 25–30% reduction in Lp(a) [31]. In other cases, Lp(a) reductions greater than 30% were not associated with the previous 2:1 LDL/Lp(a) reduction [31,32]. 

Lp(a) is mainly metabolized through the LDLR, but the affinity of Lp(a) for the LDLR is much lower than that of LDL [33]. The catabolic rate of Lp(a) was the same in FH and non-FH patients [34]. Stein et al. found that drugs increasing the cell surface number of LDLRs are ineffective on Lp(a) levels. PCSK9 inhibitors reduced Lp(a) more than LDL in homozygous familial hypercholesterolemia (HoFH) in the absence of the LDLR [35]. Identical Lp(a) levels have been found in individuals with loss-of-function and non-loss-of-function mutations of PCSK9 [36,37,38]. Epidemiological studies have not consistently confirmed the relationship between plasma PCSK9 and Lp(a) [39]. siRNA therapy inhibits the production of the apo(a) protein intracellularly at the level of translation. Its effect has a longer duration as an injection used twice a year can reduce LDL-C concentration by about 50%. Indeed, LDL-C levels decreased by 47.9%, and Lp(a) values fell by 17.2% in patients with heterozygous familial hypercholesterolemia (HeFH), according to the ORION-9 trial [40]. According to the subsequent ORION-10 study, inclisiran therapy resulted in a 25.6% decrease in Lp(a) levels in patients with cardiovascular disease [41]. The ORION-11 study indicated an 18.6% reduction of Lp(a) concentrations in individuals with cardiovascular disease or with coronary artery disease risk-equivalents [41]. These results indicate that PCSK9 inhibitor treatment, either with monoclonal antibodies or siRNA, effectively reduces Lp(a) levels and may serve as a potential adjunct to other Lp(a) lowering therapies.

## 3. LDL Production Inhibitors

Since the apo(a) protein binds to LDL particles and forms the Lp(a) lipid fraction, the question of how drugs that inhibit the production of LDL affect the Lp(a) level is involuntarily raised. Mipomersen, an antisense oligonucleotide (ASO), inhibits the synthesis of apolipoprotein B100 (apoB100), thus reducing very low-density lipoprotein (VLDL) production and leading to decreased LDL formation. Indeed, mipomersen significantly decreases LDL-C, apoB100, non-HDL cholesterol, and Lp(a) values [42]. Based on the data of four randomized double-blind phase 3 studies, mipomersen reduced circulating concentrations of Lp(a) by 26.4% in patients with various disorders of lipid metabolism and cardiovascular risk [43]. Although the exact mechanism by which mipomersen modulates Lp(a) level is still unknown, it is hypothesized that the newly synthesized apoB lipoprotein particles, to which apo(a) binds, play an important role in determining the Lp(a) levels [44].

The microsomal transfer protein (MTP) inhibitor lomitapide prevents triglyceride binding to apoB48 or apoB100, blocking intestinal chylomicron formation and hepatic VLDL synthesis. According to a study by Samaha et al., lomitapide monotherapy resulted in a 30% reduction in LDL and a 17% reduction in Lp(a) [45]. When administered to HoFH patients, lomitapide reduced Lp(a) levels by 15% [46].

## 4. Other Treatment Options to Reduce Lp(a) Concentration

### 4.1. Cholesteryl Ester Transfer Protein (CETP) Inhibitors

Upon binding to HDL, CETP promotes the transfer of cholesterol ester from HDL to the triglyceride-rich lipid particles and transfers triglyceride from the triglyceride-rich particles back to HDL, resulting in altered reverse cholesterol transport and affecting the breakdown of LDL, VLDL, and chylomicrons. In a randomized, double-blind, placebo-controlled trial, Cannon et al. found a sustained 38.8% reduction in Lp(a) levels in patients with cardiovascular disease when using anacetrapib [47]. LDL-C and Lp(a) concentrations also decreased significantly during CETP inhibitor monotherapy or in combination with a statin [48]. Later, another CETP inhibitor, namely obicetrapib (formerly TA-8995), was developed to reduce the serum levels of apoB-containing lipoproteins. Crystallography experiments showed that the potency of obicetrapib comes from its specific structure located at the narrow N-terminal neck of the hydrophobic tunnel of CETP, restricting the lipid flow [49,50]. Although these interactions between the CETP inhibitor and the opening of the tunnel are hydrophobic, three polar residues are found in the center of the inhibitor-binding site. Thus, compared to other CETP inhibitors, obicetrapib is more hydrophilic and inhibits the activity of CETP by up to 97%, raising the HDL-C level [51]. CETP inhibitors can uniformly reduce the levels of apoB-containing lipoproteins, i.e., LDL and Lp(a), in the circulation, mitigating the risk of major adverse cardiovascular events [49]. According to the data of the ROSE study, obicetrapib therapy was also found to decrease Lp(a) levels in a dose-dependent manner [52].

### 4.2. Nicotinic Acid

Nicotinic acid (niacin) is one of the oldest lipid-lowering drugs on the market with a proven Lp(a) reducing effect. Its mechanisms of action include inhibiting apo(a) transcription and curbing triglyceride synthesis and subsequent apoB secretion [53,54]. Extended-release niacin treatment resulted in a 50% decrease in the newly synthesized apo(a); however, due to the reduced catabolism, serum Lp(a) levels were only reduced by 20% [55]. In a meta-analysis of 14 randomized placebo-controlled trials, niacin was found to eventuate a 20–30% decrease in the Lp(a) concentrations [56]. In contrast, Lp(a) levels were only reduced by 21% after three years of niacin + simvastatin + ezetimibe combination therapy during the AIM-HIGH trial [57]. This relatively moderate effect may be explained by the potential apo(a) increasing effect of the statins, thus blunting Lp(a) level reduction by niacin. Based on the study by Cenarro et al., niacin induced a greater decrease in Lp(a) levels in subjects with high baseline Lp(a) levels [58], confirming the similar results of the AIM-HIGH trial [59]. The European Atherosclerosis Society recommended nicotinic acid to achieve a desirable Lp(a) level of less than 50 mg/dL in patients with moderate to high cardiovascular risk, based on the data of a meta-analysis [60].

### 4.3. Aspirin

Showing a 90% homology to plasminogen, Lp(a) binds to the plasminogen receptor and inhibits the transformation of plasminogen to plasmin and increases the production of plasminogen activator inhibitor-1, ultimately leading to increased coagulability and thrombogenicity. In the investigation of patients with atherosclerotic coronary artery disease or cerebral infarction, aspirin administration resulted in an 80% decrease in Lp(a) levels in individuals with higher initial concentrations of Lp(a) exceeding 300 mg/dL, while it did not change significantly in patients with Lp(a) levels initially lower than 300 mg/dL [61]. These findings may partly be due to the various maturation and secretion rates of the different apo(a) isoforms, as the high molecular weight isoform resides longer in the endoplasmic reticulum and has a lower secretion rate compared to the smaller isoform [62]. Aspirin is also known to inhibit apo(a) mRNA expression and transcriptional activity of human liver cell cultures and HepG2 hepatoma cells [63,64]. Aspirin and sodium salicylate inhibit nuclear factor kappa-light-chain-enhancer of activated B cells (NF-κB) and activator protein-1 (AP1) activation, therefore apo(a) gene transcription, as well [64].

Besides its proven benefits and uniform use in secondary cardiovascular prevention, aspirin is not generally recommended for primary prevention in subjects with high Lp(a) levels [65]. In the Women’s Health Study, daily administration of 100 mg aspirin reduced cardiovascular events more than 2-fold among LPA SNP rs3789220 carriers (HR, 0.44; 95% CI, 0.20–0.94), whereas the risk reduction was only modest in non-carriers (HR, 0.91; 95% CI, 0.77–1.08) [66]. It is important to mention that this study did not define the risk/benefit ratio, while the ASPREE trial found that aspirin therapy resulted in no significant net benefit due to the equally reduced prevalence of major adverse cardiovascular events and increased chance of clinically significant bleeding. However, in the rs3798220-C carrier group and the highest quintile of a Lp(a) genomic risk score distribution, aspirin reduced major adverse cardiovascular events by 11.4 and 3.3 events, respectively, without significantly increased bleeding risk, indicating a shift toward the net benefit of aspirin [67]. These data indicate that personalized medicine and assessment of the risk/benefit ratio in an educated patient is likely the best approach until further evidence is obtained for an overall net benefit in the reduction of cardiovascular events with minimal bleeding risk [65].

### 4.4. Thyroid Hormone Mimetics

Modulating lipid metabolism and thyroid hormones promotes lipolysis, enhances the production of LDL receptors, and enhances reverse cholesterol transport, increasing HDL-C levels. Vice versa, an increase in Lp(a) levels can be observed in subclinical hypothyroidism [68]. The thyroid hormone receptor agonist eprotirome reduced the Lp(a) level by 40% [69]; however, it was withdrawn due to its toxicity during long-term administration. A newer formulation, named sobetirome, produced significant LDL-C lowering and 20–40% Lp(a) lowering effects in monkeys [70].

### 4.5. Lipoprotein Apheresis

If the treatment with the above-mentioned drugs fails to achieve the target lipid values, especially in extreme hyperlipidemia, lipoprotein apheresis may be of help. Initially, Thompson et al. reported a reduction in LDL-C levels of HoFH patients after plasma exchange [71]. Currently, many national guidelines recommend lipoprotein apheresis in patients with elevated LDL-C and/or Lp(a) levels. Indeed, Lp(a) concentrations may be decreased by 60 to 90% after such intervention, leading to a 94% reduction in major cardiovascular events over 48 months, even independently from LDL-C reduction [72]. A Mendelian randomization analysis indicated that a 65.7 (95% CV: 46.3–88.3) mg/dL Lp(a) lowering would be required by a specific therapy targeting Lp(a) to reach the same potential effect on clinical outcomes as a 1 mmol/l therapeutic reduction of LDL-C levels [73]. This estimate is similar to the calculations from a substudy of HPS2-THRIVE [74]; however, other studies indicated a 99.8 (95% CV: 69.8–132.4) mg/dL reduction of Lp(a) to reach the same efficacy [67]. Data from the recently finished MultiSELECt study may provide additional information regarding the effect of lipoprotein apheresis on Lp(a) reduction and subsequent cardiovascular outcomes [75]. Also, repetitive apheresis treatments may reduce the average Lp(a) values by 50% between each treatment session, suggesting the efficacy of this therapeutic option in terms of Lp(a) reduction [76]. It also must be mentioned that lipoprotein apheresis is a costly intervention requiring special personnel and equipment and increased patient compliance, thus increasing the need for other effective treatment options.

### 4.6. Apo(a)-Reducing ASO and SiRNA Formulations

The recently developed hepatocyte-directed antisense oligonucleotides target Lp(a) mRNA and result in decreased apo(a) protein formation. Pelacarsen is a member of this group of drugs, which may lead to an 80% reduction of Lp(a) levels in patients with cardiovascular disease in a dose-dependent manner. Besides its beneficial effects on the levels of oxidized phospholipids, no serious side effects were observed [77]. The currently ongoing phase 3 Lp(a) HORIZON (Assessing the Impact of Lipoprotein(a) Lowering with TQJ230 on Major Cardiovascular Events in Patients With CVD) trial examines ASCVD risk in patients on standard LDL-lowering therapy with former cardiovascular event and 70 mg/dL or higher serum Lp(a) levels [78]. 

SLN360 is a GalNAc-conjugated siRNA that targets the LPA gene. During a single ascending dose study with SLN360, a dose-dependent reduction of Lp(a) levels was found, ranging from 40 to 98% [79]. Olpasiran is another siRNA molecule, which also proved its efficacy in reducing Lp(a) concentrations according to the data of the randomized, multicenter, double-blind, placebo-controlled trial named OCEAN[a]-DOSE (Olpasiran Trials of Cardiovascular Events and Lipoprotein[a] Reduction–Dose Finding Study). Lp(a) levels decreased in a dose-dependent manner, resulting in placebo-adjusted mean percent changes in −70.5% with the 10 mg dose, −97.4% with the 75 mg dose, −101.1% with the 225 mg dose administered every 12 weeks, and −100.5% with the 225 mg dose administered every 24 weeks. These data suggest that effective Lp(a) reduction can be achieved even if siRNA therapy is used less frequently [80]. Ongoing and future studies are required to provide evidence of whether decreased Lp(a) levels would result in reduced cardiovascular risk in patients treated with ASO or siRNA therapy [81].

Including patients over 40 years of age with Lp(a) levels above 175 nmol/L (70 mg/dL), an investigation is underway with the siRNA LY 3819469 to test changes in Lp(a) levels and side effects. The results of this study are expected by 2024 [82]. The recently available therapeutic approaches that reduce the circulating levels of Lp(a) are summarized in Table 1.

## 5. Future Perspectives

Clustered regularly interspaced short palindromic repeats (CRISPR)-Cas9 is a promising genetic approach recently emerging as a potentially effective treatment against hyperlipidemia. Compared to the other editing approaches, due to its higher specificity and efficacy, CRISPR/Cas9 has been thoroughly investigated to reduce Lp(a) levels and target the PCSK9 and LPA genes [90]. A single infusion was found to knock down PCSK9 in almost every hepatocyte of cynomolgus monkeys and lowered LDL-C by up to 60% for a period of 8 months. Results were verified in a different in vivo study with nonhuman primates, as well [91]. Designed to block PCSK9 in patients with HeFH, atherosclerotic cardiovascular disease, and uncontrolled hypercholesterolemia, this treatment option is currently being tested in a phase 1 clinical trial evaluating the safety of VERVE-101, a CRISPR base editing drug assembled by a messenger RNA for an adenine base editor and a guide RNA [92].

A novel small interfering RNA, lepodisiran (LY3819469), is also currently being tested in a phase 2 clinical trial (NCT05565742); however, human data have not yet been published [93]. Moreover, very recent results from the first-in-human phase 1 trial (NCT05565742) with muvalaplin (previously LY3473329) reported a promising 65% reduction of circulating Lp(a) levels. Muvalaplin is a selective small molecule inhibitor that binds to the kringle domains 7 and 8 of apo(a), thus preventing the covalent binding of apo(a) with apoB100 [94].

## 6. Conclusions

Several treatment options with various effects exist for Lp(a) lowering in patients with high cardiovascular risk. Inhibiting apo(a) protein synthesis at the transcriptional level, the recently developed ASOs and siRNAs are the most effective in decreasing Lp(a). Indeed, pelacarsen and olpasiran may lead to a reduction of Lp(a) levels exceeding 80%. These therapeutic options may ensure that a significant number of patients can achieve the desired Lp(a) values in the future. However, the high costs of the novel strategies and the restricted access to reimbursement in many countries might limit the efficacy of lipid-lowering treatment. Potentially replacing lipoprotein apheresis or, at least, using the novel approaches in combination with the extracorporeal Lp(a) removal, physicians may ensure attainment of the appropriate Lp(a) levels to prevent the progression of cardiovascular diseases and aortic stenosis.

## Figures and Tables

**Table 1:** Effect of lipid-lowering drugs on the concentration of circulating lipoprotein(a).

Drugs	Effect on the Concentration of Lipoprotein(a)
Statins	−13% decrease (95% CI 10–15%) in the CARDS study [16]; −15% decrease (95% CI 13–17%) in the 4S study [17]; No significant effect [83]
Ezetimibe	No effect [22]
Niacin	−22.9% dose-independent decrease (95% CI 18.5–22.9%) [56]
PCSK9 inhibitors	−26.9% decrease in FOURIER study (evolocumab) [5] −25.6% decrease in all phase 3 studies (alirocumab) [84] These data are also confirmed in meta-analyses [85]
Inclisiran	−18.6% decrease in ORION-11 study [86]
Mipomersen	−26.4% in phase 3 studies [43]
CETP inhibitors	Up to −40% decrease in a phase 2 study (evacetrapib) [87]; −34.1% decrease in a phase 2 study (anacetrapib) [88]; −33.8% decrease (5 mg/day obicetrapib) and −56.5% decrease (10 mg/day obicetrapib) [52]
Lp(a) lowering ASO and siRNA formulations	−80% decrease (pelacarsen 20 mg/week) and −72% decrease (pelacarsen 60mg/month) [89]; Placebo-adjusted −70.5% decrease (olpasiran 10 mg/12 weeks); −97.4% decrease (olpasiran 225mg/12 weeks); −100.5% decrease (olpasiran 225 mg/24 weeks [80]; Up to 86–95% decrease (SLA340 middle dose) and −98% decrease (SLA340 high dose) [79]

Abbreviations: ASO, antisense oligonucleotide; CETP, cholesteryl ester transfer protein; Lp(a), lipoprotein(a); PCSK9, proprotein convertase subtilisin/kexin type 9; siRNA, small interfering ribonucleic acid.
